# A New Hope: Sodium-Glucose Cotransporter-2 Inhibition to Prevent Atrial Fibrillation

**DOI:** 10.3390/jcdd9080236

**Published:** 2022-07-26

**Authors:** Nikolaos Karamichalakis, Vasileios Kolovos, Ioannis Paraskevaidis, Elias Tsougos

**Affiliations:** 6th Department of Cardiology, Hygeia Hospital, 15123 Athens, Greece; drkolovos@gmail.com (V.K.); iparas@otenet.gr (I.P.); tsougos@yahoo.com (E.T.)

**Keywords:** atrial fibrillation, atrial flutter, diabetes mellitus, sodium-glucose cotransporter-2 inhibitors

## Abstract

Atrial arrhythmias are common in patients with diabetes mellitus (DM), and despite recent advances in pharmaceutical and invasive treatments, atrial fibrillation (AF) and atrial flutter (AFl) are still associated with substantial mortality and morbidity. Clinical trial data imply a protective effect of sodium-glucose cotransporter-2 inhibitors (SGLT2is) on the occurrence of AF and AFl. This review summarizes the state of knowledge regarding DM-mediated mechanisms responsible for AF genesis and recurrence but also discusses the recent data from experimental studies, published trials and metanalyses.

## 1. Introduction

Patients with diabetes mellitus (DM) are constantly increasing in numbers and are estimated to reach 580 million worldwide in a 10-year projection. Diabetes mellitus induces intra-cardiac processes such as left ventricular hypertrophy (LVH), endothelium dysfunction, interstitial fibrosis, inflammation and microvascular damage and significantly impacts treating cardiovascular patients [1,2].

The occurrence of atrial fibrillation (AF) is strongly connected with DM, and AF manifestation in diabetic patients signifies unfavorable cardiovascular outcomes [3]. AF is the most popular sustained cardiac arrhythmia and is related to increased morbidity and mortality, standing as one of the primary causes of stroke and heart failure (HF) onset [4]. The reciprocal effect of DM and AF and their respective outcomes has been a matter of discussion for a long time, and the quest for therapeutic options in both diseases is more challenging than ever. Conventional antiarrhythmic drugs, acting as ion blockers, are ineffective in about half of patients suffering from AF and are associated with severe cardiac and extracardiac side effects [5]. Because of their pleiotropic actions, it is suggested that antidiabetic agents may have a role in AF inhibition. However, thus far, no glucose-lowering treatment has proven such a benefit, and results from corresponding studies with antidiabetic agents are conflicting [6].

Sodium-glucose cotransporter-2 inhibitors (SGLT2is) are one of the latest antidiabetic treatments. They have shown a broad class effect in reducing HF hospitalizations in patients with or without baseline cardiovascular disease (CVD), as well as a proven benefit in renal protection [7,8].

SGLT2is promotes glycosuria and natriuresis by restraining glucose and Na^+^ reabsorption. They also produce a reduction in blood glucose and HbA1c levels, arterial blood pressure, and body weight loss, which are critical factors for CVD and HF [9]. Because of the abovementioned mechanisms and others yet to be discovered, they have demonstrated a favorable outcome in HF patients with or without DM [10,11]. In one of the major SGLT2i cardiovascular outcome trials (CVOT), dapagliflozin decreased the incidence of reported episodes of AF-adverse events in high-risk DM patients, but it remains to be shown whether these effects apply to the class of SGLT2is [12]. In this review, we aim to address the SGLT2i mechanisms responsible for AF prevention in patients with or without DM and to discuss the published data from basic science, experimental studies, SGLT2i CVOTs and metanalyses.

## 2. Diabetes Mellitus-Related Pathophysiological Mechanisms Generating Structural and Electrical Atrial Remodeling

Many mechanisms have been proposed to explain how diabetes may predispose to AF. Obesity, hypertension, obstructive sleep apnea, and systemic inflammation are frequently associated with DM and contribute to atrial electrical and structural remodeling [13]. In a subset analysis of the AFFIRM multicenter randomized trial, AF recurrence, as measured by several attempted cardioversions and AF burden, in 2518 subjects with BMI data was found to be strongly correlated with obesity, age, hypertension and left atrial size [14]. In a Japanese prospective cohort study of 28,449 participants without baseline AF, who were followed over a mean of 4.5 years, obesity with a BMI ≥ 25 was associated with an increased risk of AF development (adjusted HR 1.64) [15]. In the same cohort, it was found that increased risk of AF manifestation was also correlated with other elements of the metabolic syndrome, such as hypertension, elevated low high-density (LDL) cholesterol, and impaired fasting glucose [15]. Obstructive sleep apnea (OSA) promotes sympathetic nervous system activation, nocturnal hypoxia as well as retention of carbon dioxide. Several observational studies in the late 1990s demonstrated a higher prevalence of AF in patients with OSA and concomitant HF or CVD [16,17]. Moreover, treatment for OSA with CPAP reduced the rate of AF recurrence by 60%, in a prospective study of 153 patients undergoing AF catheter ablation [18].

In patients with diabetes, left atrial dilatation and interstitial fibrosis represent the major trigger factor for the development of AF. Chronic inflammation and oxidative stress, increased production of advanced glycation end products (AGE), increased expression of transforming growth factor-β and changes in the expression of the gap are possible causes for the development of atrial fibrosis [19]. Advanced glycation end-products are hyperglycemia-generated and induced by oxidative stress, inflammation, renal failure and ageing. The connection of AGEs, type I collagen and elastin in the extracellular matrix in diabetic hearts leads to atrial fibrosis [20,21]. In addition, the connection of AGEs to their transmembrane receptor, the receptor of advanced glycation end-product (RAGE), impels oxidative and inflammatory procedures that favor the onset of AF [22,23]. In AF, plasma AGEs and RAGE levels are considerably higher, both in patients with and without DM, compared to those in sinus rhythm [23,24]. Atrial fibroblasts from patients with type 2 DM, compared to individuals without diabetes, have an inherent profibrotic phenotype and express higher levels of type I collagen, which may explain the augmented cardiac fibrosis and the induction of interatrial conduction delay that can enhance AF occurrence [13,25].

In a Framingham study with 2623 participants, it was shown that higher insulin resistance and hyperglycemia was associated with increased left ventricular mass [26]. In another retrospective analysis from Japan, with 4014 patients, it was found that left ventricular hypertrophy was associated with impaired left ventricular function, increased left atrial size and high AF prevalence [27].

Characteristic features of electrical remodeling in the diabetic atrium include electrical conduction disturbances and heterogeneity, action potential duration (APD) prolongation, frequency-dependent shortening of APD, increased spatial dispersion, and increased incidence of APD alternans [28]. Alterations in atrial action potential morphology, changes in conduction velocity, or susceptibility to triggered activity in diabetic hearts may be due to changes in Na^+^, K^+^ and Ca^2+^ currents [13]. In an animal study, reduced expression of ion channel proteins Kv4.3, Kv1.5 and Cav1.2 and decreased Na^+^ current have been observed in the diabetic atria [29,30]. In an atrial action potential analysis, AF has been associated with decreased L-type Ca^2+^ current (ICa-L) [31]. Gap junctions, composed of connexin (Cx) proteins (Cx40 and Cx43 are the predominant isoforms in the atrial myocardium), play a crucial role in electrical conduction in the heart [32]. In diabetic animal studies, altered Cx expression was commonly found in the atria [28]. Cxs expression and distribution are affected by DM, resulting in atrial conduction abnormalities and the promotion of AF development.

## 3. Myocardial Energetics in DM and Their Effect on AF Genesis

Myocardial energy metabolism depends strongly on fatty acids and glucose. Fatty acid metabolism is more oxygen-consuming and less efficient for ATP production compared to glucose metabolism. In people with diabetes, there is increased fatty acid metabolism and decreased glucose uptake due to insulin resistance, and the diabetic heart is prone to ischemia because of its constrained metabolic pathway [33,34]. The upscale in fatty acid metabolism causes deterioration in mitochondrial structure and decreased mitochondrial oxidative phosphorylation, promoting toxic lipid metabolite attendance and reactive oxygen species (ROS) generation. In addition, atrial mitochondria in diabetic patients are characterized by decreased respiration capacity and increased oxidative stress [35]. Moreover, mitochondrial biogenesis-related protein levels, such as peroxisome proliferator-activated receptor gamma coactivator 1-alpha (PGC-1a), nuclear respiratory factor 1 (NRF-1) and transcription factor A (Tfam), are reduced in DM [36]. The combination of these and several other pathological mechanisms leads to mitochondrial dysfunction that will eventually compromise cardiac ATP generation, promoting contractile dysfunction and arrhythmogenesis [37].

Adenosine monophosphate-activated protein kinase (AMPK) acts against metabolic stress by elevating ATP levels in cardiomyocytes via a rise in fatty acid and glucose metabolism [38]. In diabetic patients, impaired AMPK activity and atrial calcium homeostasis trigger AF onset and recurrence. On the other hand, smooth AMPK activation helps atrial calcium homeostasis and may protect against metabolic stress and AF progression [39]. Moreover, diabetic hearts are characterized by increased epicardial adipose tissue infiltration, another factor linked positively with AF [13].

## 4. Autonomic Dysfunction in DM and AF Genesis

Abnormal autonomic innervation is well recognized as an important mechanism of AF development and progression. Autonomic nervous system (ANS) disturbances can induce major changes in atrial electrophysiology and induce atrial tachyarrhythmias, such as AF [40,41]. In AF, simultaneous sympathetic and parasympathetic activations are the most common trigger for arrhythmogenesis. The onset of AF results from this imbalance between the two arms of cardiac ANS [41]. The pivotal role of the autonomic nervous system in atrial arrhythmogenesis is also supported by circadian variation in people suffering from symptomatic AF [42]. A strong association was also found between autonomic dysfunction and subclinical atrial fibrillation in patients with type 2 DM who underwent 48 h ambulatory ECG monitoring [43]. Heart rate recovery, an index of cardiac autonomic neuropathy, has been regarded as another predictor of AF risk in type 2 DM patients [44]. Regardless of the unexplored underlying cellular mechanisms, all the above findings suggest a strong link between autonomic remodeling and the occurrence of AF in patients with diabetes, considering the significant impact that the cardiac ANS has on cardiac electrophysiology. 

## 5. Effect of SGLT2 Inhibition in Atrial Fibrillation: Basic Science and Experimental Data

Mechanisms responsible for the SGLT2i benefit in AF prevention and reduction include plasma volume reduction, cardiac remodeling and enhanced cardiac energy status by increased ketone oxidation and cardio-myocyte Na–H exchange [45].

Incoming data from basic science and animal models demonstrate an interaction between SGLT2is and the atrial myocardium. Reactive oxygen species, originating from mitochondrial dysfunction, are pro-arrhythmic and have been related to AF onset and progression. Fibrosis and hypertrophy in cardiac myocytes are common in atrial fibrillation and are also linked with reactive oxygen species [19]. In a study by Yurista et al., SGLT2is have shown the capacity to elevate mitochondrial biogenesis and empower mitochondrial function [46]. Their study investigated the association between mitochondrial dysfunction and the susceptibility to develop AF and also demonstrated that empagliflozin potentially restores mitochondrial function, thus ameliorating electrical and structural remodeling. In another relevant study by Shao et al., empagliflozin had a beneficial impact in atrial structural and electrical remodeling by improving mitochondrial function and mitochondrial biogenesis in Type 2 DM, acting as a prevention agent of DM-related atrial fibrillation [36]. Administration of empagliflozin in diabetic rats significantly prevented the development of atrial myopathy and improved atrial mitochondrial respiratory function and biogenesis in DM rats [36].

SGLT2i acts in the renal proximal convoluted tubules, halting sodium/glucose reabsorption, and thus increases glucose excretion in the urine and lowers blood glucose levels but also generate natriuresis and diuresis, and eventually a reduction in atrial volume [12]. Increased uric acid levels have been related to AF onset and progression, and SGLT2is impel a favorable outcome against AF because of a reduction in uric acid plasma levels [47,48]. Hypomagnesemia is linked to AF as it increases sinus node automaticity and supraventricular ectopy [49]. SGLT2is preserve serum magnesium levels as they improve insulin sensitivity and the insulin/glucagon ratio and avoid hypomagnesemia [50]. Insulin shifts extracellular magnesium intracellularly, reducing the circulating magnesium in individuals with or without diabetes. Increased serum magnesium levels can also generate an anti-ischemic and anti-inflammatory effect on the heart [51].

In addition, SGLT2is have been shown to produce a beneficial reduction in epicardial fat. This dynamic pathogenic tissue has been related to coronary artery disease but also increased AF incidence and severity [13,52]. In their study, Sato et al. demonstrated that patients who received dapagliflozin presented significant epicardial fat volume reduction at a six-month follow-up compared with the control group [53].

SGLT2is also decrease obesity, systemic inflammation, oxidative stress and sympathetic overdrive, which are important factors for AF onset and progression [9,40,54].

Glycemic variations, namely hypoglycemia, have been linked to an increased risk of AF in DM patients [55]. The hypoglycemia risk in SGLT2i is negligible compared to other glucose-lowering agents, thus triggering AF events caused by glycemic variations are avoided in patients receiving SGLT2i. The glycemic lowering of SGLT2i is insulin independent and related to decreased glucose reabsorption in the kidney and the risk of hypoglycemia with SGLT2i is minimal in contrast to other agents, such as insulin or sulphonylureas, that can cause hypoglycemia [56,57].

In the EMPA-HEART study, 97 DM patients who received empagliflozin demonstrated a reduction in left ventricular (LV) mass, as measured by cardiac magnetic resonance imaging after a follow-up of six months. Along with the improvement in LV mass indexed to body surface area, the empagliflozin group exhibited a reduction in systolic/diastolic arterial pressure and an increase in hematocrit, which may account in part for the beneficial cardiovascular outcomes [58].

In a sub-study of the EMPA-HEART study, Mazer et al. demonstrated a beneficial impact in red blood cell production induced by increased erythropoietin secretion in patients receiving empagliflozin. Erythropoietin can produce favorable hemodynamic and myocardial energetic alterations, adding an extra systematic anti-inflammatory/pro-angiogenic effect, promoting SGLT2i cardioprotection [59].

SGLT2is delivers further cardiovascular benefits in human physiology by soothing inflammasome activity. In a study by Sukhanov et al., smooth muscle cells (SMCs) from human aortas were used to investigate the role of empagliflozin in vascular inflammation. Empagliflozin mitigates NLPR3-associated overproduction of cytokines IL-1β and IL-18 as well as SMC migration and proliferation [60]. In another study by Kim et al., empagliflozin demonstrated high levels of inflammasome mitigation, an outcome strongly connected to improved metabolic factors such as lower levels of fasting serum insulin and uric acid in parallel with higher levels of serum ketone bodies. They compared NLRP3 activity in human macrophages (paracrine IL-β production and mRNA levels) between 29 patients receiving empagliflozin and 32 receiving sulfonylurea (glimepiride) for 30 days [61].

**Figure 1:**
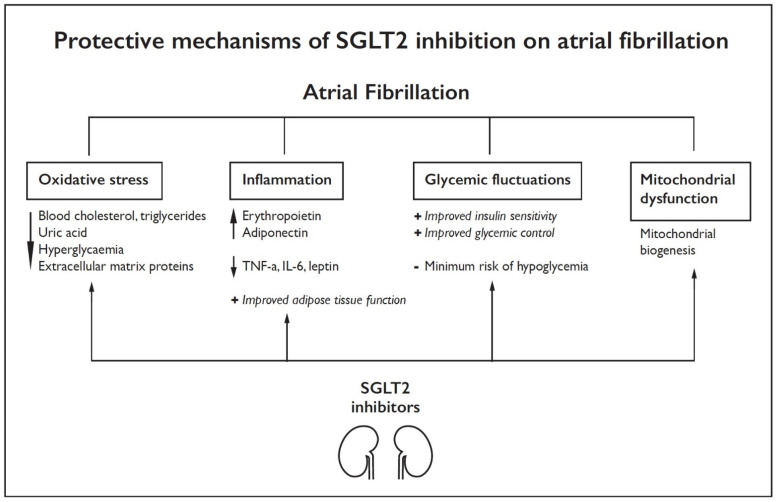
Protective mechanism of SGLT2 inhibition on atrial fibrillation. SGLT2: sodium-glucose cotransporter-2, TNF: tumor necrosis factor, IL: interleukin.

The abovementioned protective mechanisms of SGLT2i on AF are summarized schematically in Figure 1.

## 6. Effect of SGLT2 Inhibition in Atrial Fibrillation: Clinical Data and Metanalyses

In the DECLARE-TIMI 58 (Dapagliflozin Effect on Cardiovascular Events–Thrombolysis in Myocardial Infarction 58) study, 769 AF episodes occurred in 589 patients over a median follow-up of 4.2 years. A total of 124 patients had two AF episodes, 36 patients had three episodes, and 20 patients had ≥ four episodes [62]. The maximum number of episodes in a single patient was six and seven. A total of 6.5% of the participants (1116) suffered from AF at baseline, who tended to be older and to have a higher body mass index, a history of coronary artery disease and HF, and higher urine albumin-to-creatinine ratio at baseline, and lower baseline-estimated GFR [12]. All these factors contribute to AF manifestation. The risk of the first AF episode was reduced by 19% (264 versus 325 episodes; 7.8 versus 9.6 events per 1000 patient-years; hazard ratio [HR], 0.81 [95% CI, 0.68–0.95]; P = 0.009;). However, these positive results should be taken with a grain of salt. As stated by the authors, ECGs were not routinely collected nor independently reviewed, and events of AF/AFL were not centrally confirmed.

When the analysis was limited to AF episodes that met the criteria as serious adverse events (132 versus 166 episodes; HR, 0.79 [95% CI, 0.63–0.99]) and to those linked with hospitalization (114 versus 147 episodes; HR, 0.77 [95% CI, 0.60–0.98]), the dapagliflozin beneficial effect was preserved [62]. Interestingly, when the preferred terms search was extended to include “atrial tachycardia” (264 versus 326 episodes; HR, 0.80 [95% CI, 0.68–0.94]) and “supraventricular tachyarrhythmia/tachycardia” (280 versus 336 episodes; HR, 0.83 [95% CI, 0.71–0.97]) similar outcomes were noticed [12].

Dapagliflozin also reduced the total number of AF and atrial flutter (AFL) episodes (337 versus 432; incidence rate ratio, 0.77 [95% CI, 0.64–0.92]; P = 0.005). The application of the Wei–Lin–Weissfeld model revealed a treatment effect for the first episode as well as for the subsequent episode (first event: HR, 0.81 [95% CI, 0.68–0.95]; P = 0.009; second event: HR, 0.69 [95% CI, 0.49–0.99]; P = 0.045; third event: HR, 0.50 [95% CI, 0.25–0.99]; P = 0.048; average HR, 0.81 [95% CI, 0.68–0.95]; P = 0.009) [12].

In the EMPA-REG OUTCOME trial, the empagliflozin beneficial effect on HF-related outcomes such as HF hospitalizations, CV death, all-cause death, and the first administration of loop diuretics and development of edema, was consistent in both patients with AF and without AF [63,64]. On the other hand, patients with baseline AF presented a higher rate of the abovementioned events. Still, their absolute number prevented by empagliflozin was more significant than in patients with no baseline AF. In the trial, new events of AF were limited, and no difference in terms of AF onset was noticed between empagliflozin and placebo, as the prevalence of new-onset AF was 2.4% with placebo and 2.9% with empagliflozin. However, AF analysis was performed in a DM population with high CV risk and the possibility of AF prevention could be revisited in upcoming trial outcomes [64].

The CANVAS and CANVAS-R trials with canagliflozin were CV safety studies that compared SGLT2i canagliflozin with placebo and the overall prevalence of new-onset AF was 5.96% with canagliflozin against 6.04% with placebo [65,66]. When subjects were categorized based on the presence or absence of HF, there was a significantly higher prevalence of AF in HF patients (14.4% vs. 4.6%, *p* < 0.001), but AF as a risk factor did not seem to affect CV death or hospitalization for HF during the trial (p 0.47) [66,67].

In a population-based propensity score-matched cohort study consisting of 79,150 DM patients receiving SGLT2is compared with 79,150 matched DM patients receiving DDP-4 inhibitors, it was shown that there was a 17% reduction of new-onset AF in the SGLT2i arm [68]. The study aimed to evaluate the risk of new-onset arrhythmias and all-cause mortality in type 2 DM patients receiving SGLT2i [68].

The CVD REAL Nordic study enlisted T2D patients receiving antidiabetic drugs and was conducted between 2012 and 2015 in nationwide registries in Denmark, Norway and Sweden [69]. Patients were divided in two groups: (i) new users of dapagliflozin (n = 10,227) and (ii) new users of DPP-4 inhibitors (n = 30,681). New-onset AF occurred at a rate of 1.47 per 100 patient-years with dapagliflozin and 1.58 episodes/100 patient-years with DPP-4 inhibitors, respectively (HR 0.92 NS) [69,70].

In another observational study by Norhammar et al., dapagliflozin was examined in a Swedish population regarding cardiovascular outcomes [71]. Data from the D360 Nordic program were collected and generated in a cohort of 7102 type-2 DM subjects. A group of 21,306 propensity-matched control patients taking other glucose-lowering medications was set as a comparison, and after a mean follow-up of 1.6 years, dapagliflozin demonstrated cardioprotective outcomes comparable to those in DECLARE TIMI 58. A statistically significant reduction in all-cause and cardiovascular mortality was noticed, but with regard to AF episodes, no difference was demonstrated (HR 0.94; *p* = 0.425) [71].

In a study with HF patients with non-ischemic etiology and type 2 DM, SGLT2is reduced the risk of developing new-onset AF [72]. A total of 210 HF patients with non-ischemic and sinus rhythm and reduced left ventricular ejection fraction of 31.0 ± 8.2% were enrolled; 60 of them also suffered from DM. Kaplan–Meier curve analysis showed that non-ischemic HF patients without DM suffered from fewer AF occurrences compared with those with DM (log-rank *p* = 0.0003). Of the 60 HF and DM patients, those treated with SGLT2is (20 with dapagliflozin, 7 with empagliflozin and 5 with canagliflozin) experienced fewer occurrences of the development of new-onset AF compared to those not receiving SGLLT2is (log-rank *p* = 0.040). Despite the limited number of patients, it was shown that DM is related to new-onset AF manifestation and SGLT2i administration can significantly reduce AF development in DM patients [72].

The number of published metanalyses evaluating the effectiveness of SGLT2i in AF prevention is steadily increasing. In their work, Okunrintemi et al. included the four CV safety trials (EMPA-REG OUTCOME, CANVAS, DECLARE-TIMI 58 and VERTIS CV), three renal outcome trials (CANVAS-R, CREDENCE and DAPA-CKD) and one HF trial (DAPA-HF) [73]. In these trials, 0.9% (295 of the 31,261) participants who received SGLT-2i had at least one AF episode reported as a serious adverse event against 1.1% (291 of 24,705) participants in the placebo group. Metanalysis of these trials demonstrated a significantly lower incidence of AF in participants with and without DM favoring SGLT2is against placebo (RR [95% CI] =0.79 [0.67, 0.93], I^2^ = 0%) and the number needed to treat (NNT) was 427 [73].

Li et al. gathered data from 16 identified trials, including 38,335 T2D participants [74]. Metanalysis of these trials demonstrated that SGLT2 is significantly reduced AF/AFL (RR: 0.76; 95% CI 0.65–0.90; *p* = 0.001), all-cause mortality (RR: 0.91; 95% CI 0.83–0.99; P = 0.03) and HF outcomes (RR: 0.73; 95% CI 0.64–0.84; *p* < 0.00001). AF/AFL reduction related to SGLT2i was independent of age, body weight, HbA1c, or systolic blood pressure at baseline (all *p*-interactions > 0.3) [74].

In a metanalysis by Fernades et al., which included 34 randomized trials with 63,166 patients, canagliflozin, dapagliflozin, empagliflozin and ertugliflozin were investigated based on their effect on AF prevention [75]. The class of SGLT2is demonstrated a significant reduction in risk of incident atrial arrhythmias (OR, 0.81, 95% CI 0.69–0.95; P = 0.008) in patients with diabetes.

Combined data from three major SGLT2i CVOTs (the CANVAS Program, DECLARE-TIMI-58, and CREDENCE) revealed that AF/AFL events were reduced by 19% in the receiving SGLT2i group, even though that was not the primary analysis endpoint [76]. However, data from the CREDENCE sub-analysis did not support the beneficial upturn in AF/AFL incidence (HR 0.76; 95% CI Antiarrhythmic Potential of SGLT2is Antiarrhythmic Potential 1389 0.53–1.10; *p* = 0.15), setting the hypothesis that the positive result was driven by data from the DECLARE-TIMI-58 trial [77].

A recent metanalysis by Zheng et al. included 20 randomized trials involving 63,604 patients, most of them with DM. The primary analysis evaluated the incidence of AF and stroke [78]. The SGLT2 inhibitors evaluated were dapagliflozin (seven studies, 28,834 patients), empagliflozin (five studies, 9082 patients), canagliflozin (seven studies, 17,440 patients), and ertugliflozin (one study, 8246 patients). SGLT2i administration was related to a significant attenuation in the risk of AF incidence (odds ratio = 0.82; 95% confidence interval, 0.72–0.93; P = 0.002) compared with control. However no significant difference was demonstrated in stroke between SGLT2is and controls (odds ratio = 0.99; 95% confidence interval, 0.85–1.15; P = 0.908). However, given the heterogeneity of the studies analyzed, there was no standard protocol for AF assessment and AF risk factors may be mi-matched between the SGLT2i and control group, which is a major problem with all current metanalyses.

The clinical data published thus far are, without doubt, hypothesis-generating. In addition, the SGLT2i class effect on AF prevention cannot be extrapolated since current meta-analyses inconsistently clarify that data from DECLARE-TMI-58 (with dapagliflozin) predominantly affect the results in terms of AF prevention [77]. In order to prevent further confusion in clinical practice, adequately powered randomized SGLT2is trials should be performed, with primary AF endpoints, such as new-onset AF, AF recurrence/burden, in patients with or without DM, with different SGLT2i agents. Such trials, like the EMPA-AF (NCT04583813) with empagliflozin in patients with DM or obesity, HF and AF and the DAPA-AF with dapagliflozin versus placebo in patients undergoing AF catheter ablation, are ongoing.

## 7. Conclusions

The results from the abovementioned studies and analyses provide experimental and clinical data for a SGLT2i-induced favorable effect on the incidence of AF. SGLT2i agents are now validated HF drugs due to their impressive outcomes in clinical trials, not only in the DM population, but also in patients with HF or at high risk for CVD and their pleiotropic effects may provide a great benefit in reducing AF. Further research at experimental and clinical levels is required to evaluate AF manifestation and progression in well-defined populations of patients with or without DM and to explore the dynamics of SGLT2i in this process.

## Data Availability

Not applicable.
